# Dental pulp stem cells for reconstructing bone defects: A systematic review and meta-analysis

**DOI:** 10.34172/joddd.2022.034

**Published:** 2022-12-30

**Authors:** Neda Moeenzade, Mohsen Naseri, Fereshteh Osmani, Fariba Emadian Razavi

**Affiliations:** ^1^Student Research Committee, Birjand University of Medical Sciences, Birjand, Iran; ^2^Cellular and Molecular Research Center, Department of Molecular Medicine, Birjand University of Medical Sciences, Birjand, Iran; ^3^Infectious Diseases Research Center, Birjand University of Medical Sciences, Birjand, Iran; ^4^Clinical Research Development Unit, School of Dentistry, Birjand University of Medical Sciences, Birjand, Iran

**Keywords:** Bone regeneration, Dental pulp, Mesenchymal stem cells, Tissue engineering, Meta-analysis

## Abstract

**Background.:**

Bone reconstruction with appropriate quality and quantity for dental implant replacement in the alveolar ridge is a challenge in dentistry. As dental pulp stem cells (DPSCs) could be a new perspective in bone regeneration in the future, this study investigated the bone regeneration process by DPSCs.

**Methods.:**

Electronic searches for articles in the PubMed, EMBASE, and Scopus databases were completed until 21 April 2022. The most important inclusion criteria for selecting in vivo studies reporting quantitative data based on new bone volume and new bone area. The quality assessment was performed based on Cochrane’s checklist.

**Results.:**

After the title, abstract, and full-text screening of 762 studies, 23 studies were included. A meta-analysis of 70 studies that reported bone regeneration based on new bone area showed a statistically significant favorable influence on bone tissue regeneration compared to the control groups (*P*<0.00001, standardized mean difference [SMD]=2.40, 95% CI: 1.55‒3.26; I^2^=83%). Also, the meta-analysis of 14 studies that reported new bone regeneration based on bone volume showed a statistically significant favorable influence on bone tissue regeneration compared to the control groups (*P*=0.0003, SMD=1.85, 95% CI: 0.85‒2.85; I^2^=84%).

**Conclusion.:**

This systematic review indicated that DPSCs in tissue regeneration therapy significantly affected bone tissue complex regeneration. However, more and less diverse preclinical studies will enable more powerful meta-analyses in the future.

## Introduction

Reconstruction of bone defects is often a clinical challenge, especially in dentistry. Maxillofacial bone deficiencies result from tooth loss, periodontitis, trauma, tumor removal, congenital anomalies, and radiation-related osteonecrosis. Periodontitis and ridge remodeling following tooth loss is the most common cause of the alveolar bone defect. Successful implant placement requires adequate bone quality and quantity to avoid implant failure; therefore, reconstructing the alveolar ridge is a substantial issue for dental implant-supported prostheses.^1-3^

Autogenous bone grafting is the gold standard for bone regeneration. However, mitigating the complications associated with the harvest of autologous bone was the primary impetus for developing bone graft substitutes.^4^ Reconstructing bone defects with tissue engineering using dental pulp stem cells or DPSC is one of the most modern rehabilitation methods that can revolutionize future treatments.^5^

DPSCs can include self-renewal capacity, multilineage differentiation capacity, high proliferation potential, and clonogenic efficacy. These features have made them the most promising mesenchymal stem cells (MSCs) for clinical purposes. However, many issues and challenges must be addressed before using these cells in clinical treatment.^6,7^

Tissue engineering scaffolds can facilitate the proliferation and differentiation of progenitor cells. Combining osteogenic cells, osteogenic factors, biocompatible scaffolds, and angiogenesis are the elements of bone tissue engineering. Treatment with bone-related factors, gene transfection, and gene overexpression enhances the bone regeneration potential of DPSCs.^5,8^

 Due to the limited clinical trials conducted in the field of bone regeneration by DPSCs, they have not yet been effectively used in clinical treatments. Further investigation of the studies conducted in this field will lead to achieving suitable study designs and, ultimately, the progress of therapy using DPSCs. Since the quantitative evaluation of bone regeneration by DPSCs has not been carefully evaluated in the previous systematic reviews, this study aimed to evaluate the potential of DPSCs in clinical and preclinical bone regeneration from a quantitative point of view. For this purpose, this review study analyzed the amount of bone volume and bone area regenerated by DPSCs.

## Methods

###  Protocol

The Preferred Reporting Items for Systematic Reviews and Meta-Analyses (PRISMA) statement^9^ was the protocol of this systematic review.

###  Focus questions 

 The objective of this study was to review the literature to answer the focused question systematically:

 Do DPSCs improve the quantitative results of bone regeneration?

 Which samples, scaffold type, final follow-up, and defect type significantly impact bone regeneration?

###  Eligibility criteria

 The inclusion criteria for the selection were:

*In vivo* studies, bone defect regeneration therapy utilizing DPSCs, studies reporting quantitative data in the form of new bone volume percentage or new bone area percentage, and studies published in the English language.

 The exclusion criteria for the selection were:

 Studies reporting only qualitative results of bone regeneration

 In studies where the results of bone regeneration had been reported quantitatively, the exclusion criteria were these items:

 Standard deviations were not apparent, the numbers of samples were not reported, the created bone defects were not filled by scaffolds seeded with DPSCs in the test group, and an acellular scaffold did not fill the created bone defects in the control group.

###  Information sources

 Electronic searches were completed for articles in MEDLINE (PubMed), EMBASE, and Scopus databases until 21 April 2022. Also, related systematic references were added.

###  Search strategy 

 The search strategy was: “regenerate *” AND “bone*” AND (“stem” or “pulp”), AND “cell.”

###  Selection process

 The studies were evaluated by two reviewers separately (NM and FO), and the third reviewer (FE) reviewed the differences.

###  Data collection process

 Two reviewers (NM and FO) collected data from each report independently.

###  Data items

 The list and definition of outcomes are as follows:

 “Author-year” specifies the author and year of publication.

 “Sample” specifies the type of animals with bone defects.

 “Number” specifies the total number of test and control group samples.

 “Site and size of bone defects” specifies the type of the created bone defects and their dimensions or the dimensions of the bur used.

 “Final follow-up” specifies the final duration of treatment by week.

 “Laboratory method” specifies the laboratory method.

 “Scaffold” specifies the scaffold used.

 “Regenerated bone area” specifies the study outcome based on bone area percentage. The results of the test and control groups for each study are stated in this column. In the test group, the defect was filled by scaffolds seeded with DPSCs, and in the control group, the defect was supplied with an acellular scaffold.

 “Regenerated bone volume” specifies the study outcome based on bone volume percentage. The results of the test and control groups for each study are stated in this column. In the test group, the defect was filled by scaffolds seeded with DPSCs, and in the control group, the defect was supplied by an acellular scaffold.

###  Study risk of bias assessment

Cochrane’s risk of bias tool was used to assess the risk of bias in the included studies.^10^ The criteria used were as follows: random sequence generation (selection bias), allocation concealment (selection bias), blinding of personnel (performance bias), blinding of outcome assessment (detection bias), incomplete outcome data (attrition bias), selective reporting (reporting bias), and other sources of bias.

 The classification of studies based on seven criteria of risk of bias assessment was as follows:

 A study had a low risk of bias if it had none of the types of preferences, a study had a moderate risk of bias if it had one of the types of bigotry, and a study had a high risk of bias if it has more than one type of bias.

###  Statistical analysis

 Review Manager (RevMan, Computer program, Version 5.4, The Cochrane Collaboration, 2020) was used for statistical analysis. Separate meta-analyses were performed according to regenerated bone volume and area. In addition, subgroup analyses were performed according to the sample, defect, follow-up, and scaffold types.

Meta-analyses were performed using the Z test with random effects weighted inverse variance method. The effect size was measured using standardized mean differences (SMDs) and 95% confidence intervals. SMD < 0.2 was considered a ‘small’ effect size, SMD between 0.2 and 0.8 represented a ‘medium’ effect size, and SMD > 0.8 was considered a ‘large’ effect size. The results were considered significant when P < 0.05. The heterogeneity was assessed using the I^2^ test. If I^2^ was > 75%, it was interpreted as highly heterogeneous.

## Results

###  Study selection

**Figure 1:**
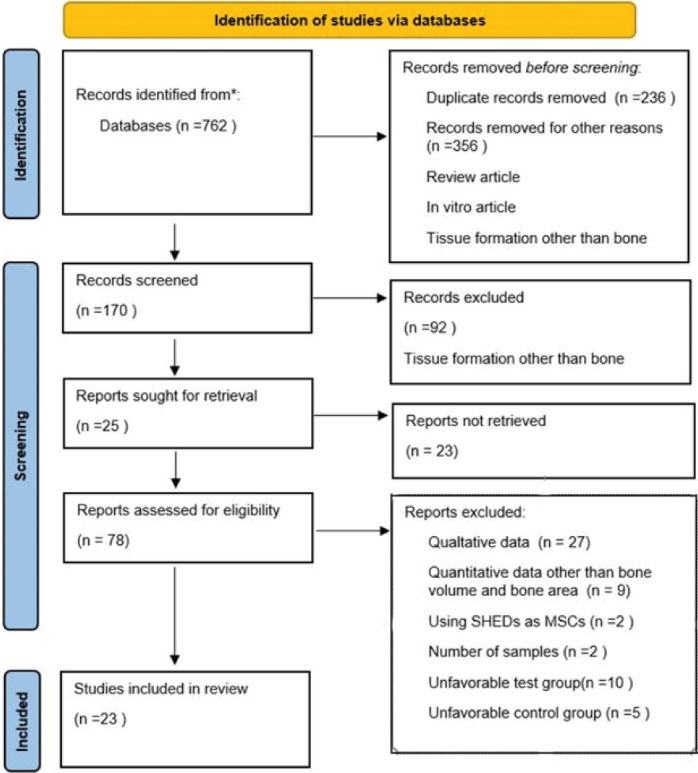
Flow diagram for included searches of databases.Abbreviations: MSCs: mesenchymal stem cells; SHEDs: stem cells from human exfoliated deciduous teeth

A total of 762 records were identified through database searches. After removing duplicate articles, in vitro articles, review articles, and articles that did not investigate bone tissue regeneration, the full texts of 78 studies were reviewed. The reasons for excluding studies after a full-text assessment were as follows: The quantitative data were not reported (n = 27)^11-37^; the percentage of new bone volume or new bone area was not reported (n = 9)^38-46^; the number of the samples was not reported (n = 2)^47,48^; stem cells from human exfoliated deciduous teeth were used as MSCs (n = 2)^49,50^; the created bone defects in the test group were not filled by scaffolds seeded with DPSCs (n = 10)^51–59^; and the created bone defects in the control group were not filled by an acellular staging (n = 5).^60-64^ Finally, 23 studies were included in the meta-analysis.^65-87^ Figure 1 shows the flow chart.

###  Study characteristics

The samples used in the studies were rats in 9 studies,^65,67,69,71,74,76,78,79,85^ mice in 5 studies,^80,81,83,84,87^ rabbits in 4 studies,^72,77,78,82^ sheep in 2 studies,^68,70^ and pigs in 2 studies.^73,86^

The bone defects were created in the cranium in 15 studies,^65,67,68-72,74,79,80,82-84,86^ in the mandible in 2 studies,^67,73^ in the alveolar bone in 4 studies,^68,69,78,86^ and in the femur in 2 studies.^70,80^ Dimensions of defect, Scaffolding used, and final follow-up varied across studies.

The results were in the form of a new bone area in 10 studies^65,68,70,71,73,74,76,78,79,84^ and new bone volume in 7 studies.^69,75,80,81,83,85,86^

Six studies reported outcomes in both forms.^66,67,72,77,82,87^ Table 1 shows the study characteristics.

**Table 1:** Classification of in vivostudies based on their features

**Author, year**	**Sample**	**Number**	**Site and size of bone defects**	**Final follow-up**	**Laboratory method**	**Scaffold**	**Regenerated bone area**	**Regenerated bone volume**
Vater et al, 2022^80^	Mice	23	Femur 15.7 mm^3^	6 Weeks	Histology µCT	MCM		T = 29.45 ± 19.5% C = 28.28 ± 12.8%
Colorado et al, 2022^65^	Rats	10	Calvarium 5 mm	10 Weeks	Histology Radiology SEM	PLGA/HA	T = 5.14 ± 0.13% C = 4.98 ± 0.16%	
Chan et al, 2022 ^77^	Rabbits	12	Calvarium 6 mm	8 Weeks	Histology Histomorphometry Immunohistochemistry µCT	HA-TCP	T = 39.78 ± 2.45% C = 38.01 ± 2.45%	T = 41.32 ± 3.57% C = 39.81 ± 3.16%
Maillard et al, 2022^81^	Mice	10	Calvarium 3.5 × 1 mm^2^	8 Weeks	Histology Histomorphometry µCT	Hydrogel		T = 11.67 ± 2.85% C = 4.84 ± 2.33%
Zhu et al, 2021^87^	Mice	18	Calvarium 2 mm	8 Weeks	Histology Radiology µCT	Collagen	T = 32.51 ± 2.46% C = 24.64 ± 2.03%	T = 55.86 ± 3.31% C = 47.56 ± 2.33%
Shiu et al, 2021^82^	Rabbits	8	Calvarium 6 mm	8 Weeks	Histology Histomorphometry µCT	MBCP (HA and tricalcium phosphate)	T = 39.8 ± 5.7% C = 38.3 ± 6.0%	T = 41.0 ± 1.4% C = 38.4 ± 1.3%
Shiu et al, 2021^82^	Rabbits	8	Calvarium 6 mm	8 Weeks	Histology Histomorphometry µCT	Bio-Oss	T = 42.1 ± 2.7% C = 41.3 ± 3.5%	T = 41.2 ± 3.4% C = 39.0 ± 5.1%
Park et al, 2020^83^	Mice	8	Calvarium 4 mm	8 Weeks	Histology Immunohistochemistry µCT	Dense collagen (S53P4)		T = 3.62 ± 1.94% C = 6.47 ± 4.08%
Jin et al, 2019^67^	Rats	10	Mandible 2 × 1 mm^2^	6 Weeks	Histology µCT	Hydrogel	T = 6.15 ± 0.55% C = 1.30 ± 0.29%	T = 26.03 ± 3.53% C = 8.95 ± 3.25%
Çolpak et al, 2019^68^	Sheep	32	Alveolar bone 3.7 × 10 mm^2^	6 Weeks	Histology Histomorphometry	Granular deproteinized bovine bone with 10% porcine collagen	T = 29 ± 1.07% C = 18.45 ± 0.33%	
Lin et al, 2019^69^	Rats	20	Alveolar bone 2 × 1.5 × 0.5 mm^3^	2 Weeks	Histology µCT	Matrigel		T = 47.61 ± 7.08% C = 30.08 ± 2.13%
Campos et al, 2019^70^	Ovine	12	Femur 5 mm	17 Weeks	Histology Histomorphometry Radiology	Bonelike® plus Tisseel Lyo®	T = 77.5 ± 3.2% C = 67.9 ± 3.9%	
Lee YC et al, 2019^66^	Rabbits	12	Calvarium 6 mm	6 Weeks	Histology Histomorphometry Immunohistochemistry µCT	Bio Oss	T = 33.5 ± 9.3% C = 25.6 ± 9.7%	T = 48.3 ± 3.0% C = 43.5 ± 0.9%
Yuan et al, 2018^71^	Rats	20	Calvarium 5 mm	12 Weeks	Histology Histomorphometry Immunohistochemistry µCT	Bio-Oss	T = 34.69 ± 4.68% C = 24.69 ± 2.44%	
Collignon et al, 2018 ^84^	Mice	12	Calvarium 3.5 mm	12 Weeks	Histology Histomorphometry µCT	Collagen	T = 65.01 ± 11.38% C = 35.25 ± 18.47%	
Wongsupa et al, 2017^72^	Rabbits	6	Calvarium 11 mm	8 Weeks	Histology Histomorphometry µCT Clinical	PCL/BCP	T = 11.36 ± 3.56% C = 6.68 ± 1.38%	T = 25.33 ± 0.61% C = 13.28 ± 2.46%
Chamieh et al, 2016^85^	Rats	15	Calvarium 5 mm	5 Weeks	Histology Histomorphometry µCT	Collagen		T = 9.86 ± 1.92% C = 3.07 ± 0.52%
Kuo et al, 2015^73^	Pigs	8	Mandible 6 mm	8 Weeks	Histology Histomorphometry	CSD	T = 69.7 ± 4.9% C = 33.9 ± 9.9%	
Cao et al, 2015^86^	Pigs	8	Alveolar bone 5 × 7 × 3 mm^3^	12 Weeks	Histology Histomorphometry Radiology Clinical	HA-TCP		T = 56 ± 3.6% C = 0.47 ± 2.19%
Petridis et al, 2015^74^	Rats	30	Calvarium 5 mm	8 Weeks	Histology Histomorphometry	Hydrogel	T = 32.78 ± 9.24% C = 24.40 ± 8.29%	
Annibali et al, 2014^75^	Mice	10	Calvarium 4 × 1 mm^2^	8 Weeks	Histology Histomorphometry	Granular deproteinized bovine bone with 10% porcine collagen		T = 17.67 ± 20.17% C = 16.21 ± 9.74%
Maraldi et al, 2013^76^	Rats	20	Calvarium 5 × 8 × 1.5 mm^3^	4 Weeks	Histology Histomorphometry Immunohistochemistry Radiology	Collagen	T = 56.80 ± 4.34% C = 43.58 ± 7.15%	
Pisciotta et al, 2012^79^	Rats	10	Calvarium 5 × 8 × 1.5 mm^3^	6 Weeks	Histology Histomorphometry Immunohistochemistry	Collagen	T = 69.03 ± 7.87% C = 39.21 ± 4.36%	
Liu et al, 2011^78^	Rabbits	12	Alveolar bone 10 × 4 × 3 mm^3^	12 Weeks	Histology Histomorphometry Radiology	nHAC/PLA	T = 35.95 ± 2.53% C = 22.86 ± 0.55%	

T: test group; C: control group; µCT: X-ray micro-computed tomography; SEM, Scanning electron microscopy; MCM, Mineralized collagen matrix; HA-TCP, Hydroxyapatite /Tricalcium phosphate; HA, hydroxyapatite; PLGA, Polylactide-co-glycolide; CSD, Calcium sulfate dehydrate; PCL/BCP, polycaprolactone/β-tricalcium phosphate; HA, Hydroxyapatite; TCP, Tricalcium phosphate; nHAC, Nanohydroxyapatite/ collagen; PLA, poly(L-lactide).

###  Risk of bias in studies

**Figure 2:**
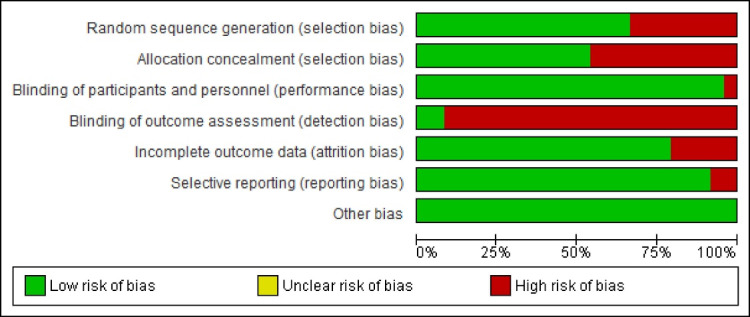
Risk of bias graph: reviewing authors’ judgments about each risk of bias item presented as percentages across all included studies

**Figure 3:**
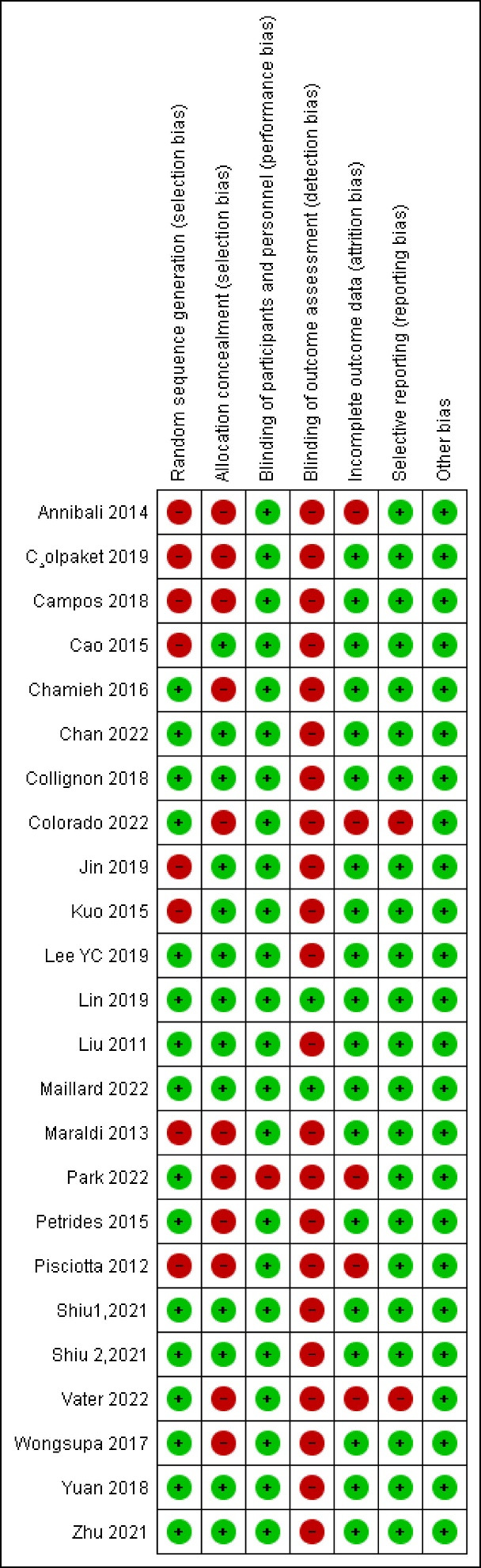
Risk of bias summary: reviewing authors’ judgments about each risk of bias item for each included study

In this category, 2, 8, and 13 articles showed a low, medium, and high risk of bias, respectively. Figures 2 and 3 show reviewing authors’ judgments about each risk of bias item presented.

###  Analysis based on new bone area percentage

**Figure 4:**
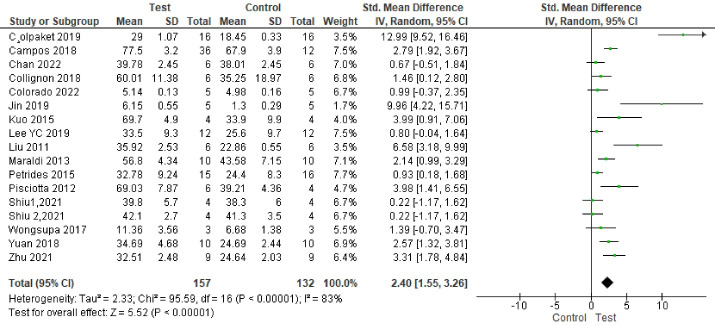
Forest plot for new bone area measures

**Figure 5:**
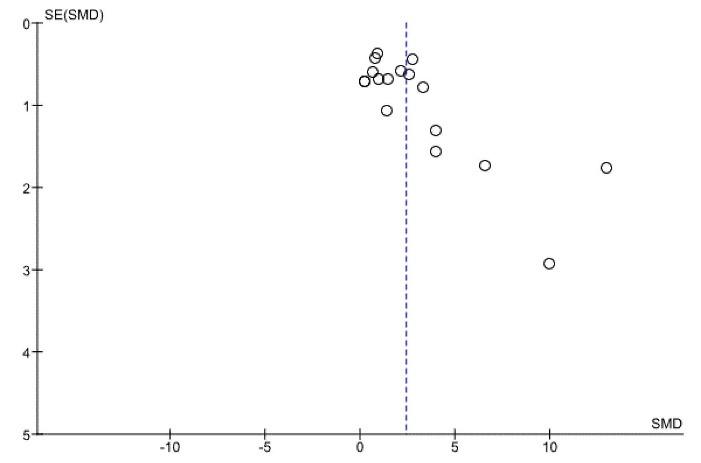
Funnel plot for new bone area measures

Seventeen studies reported results based on bone area percentage.^65,66,67,69,71,74–82,84–86^ Study results were highly heterogeneous. Bone formation was significantly enhanced in the test groups compared to the control groups (*P* < 0.00001, SMD = 2.40, 95% CI: 1.55‒3.26; participants = 289; studies = 17; I^2^ = 83%) (Figures 4 and 5).

**Figure 6:**
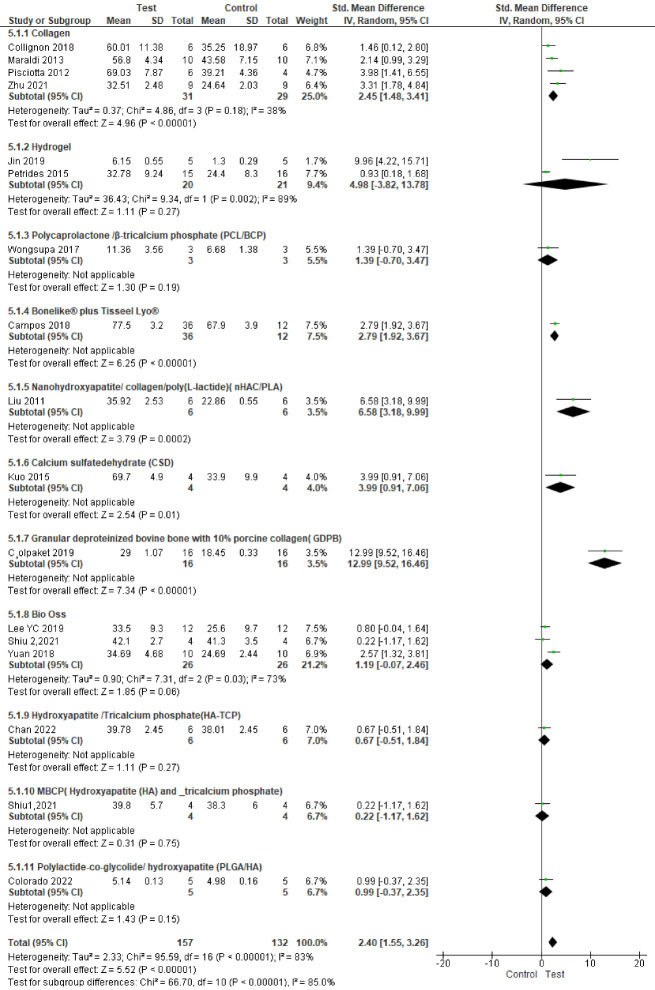
Forest plot for new bone area measures stratified by scaffold type

**Figure 7:**
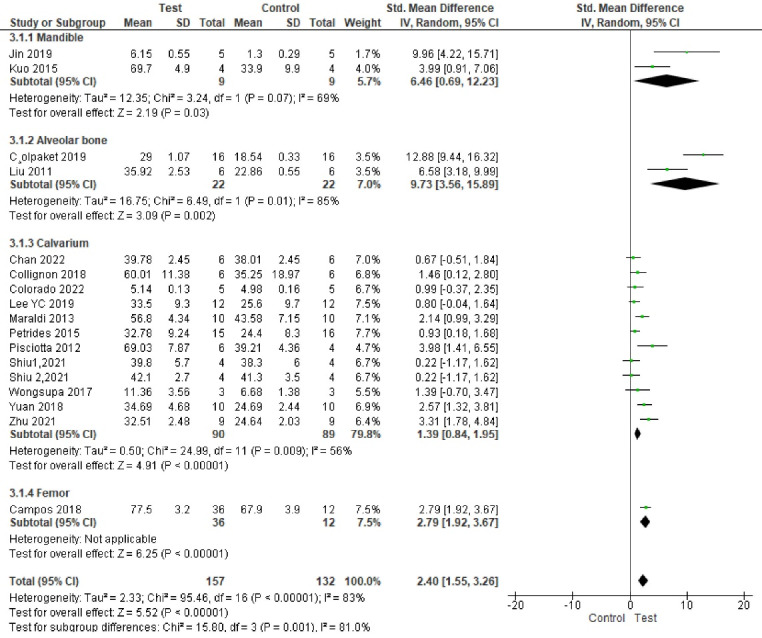
Forest plot for new bone area measures stratified by defect type

Subgroup analyses showed a statistically significant difference in bone regeneration with different scaffold and defect types. The granular deproteinized bovine bone group enhanced bone regeneration the most (SMD = 12.99 [95% CI: 9.52‒16.46]). However, only one study formed this subgroup, and thus this result has low statistical power (Figure 6). The alveolar bone defect subgroup had the biggest and significantly different effect size compared to other defect types (Figure 7 ). The SMDs in the alveolar and mandibular bone defect subgroup were 9.73 ([95% CI: 3.65‒15.89], studies = 2) and 6.46 ([95% CI: 0.69‒12.23], studies = 2), respectively, higher than that in the calvarium bone defect subgroup (SMD = 1.39 [95% CI: 0.84‒1.95], studies = 12) (Figure 7).

**Figure 8:**
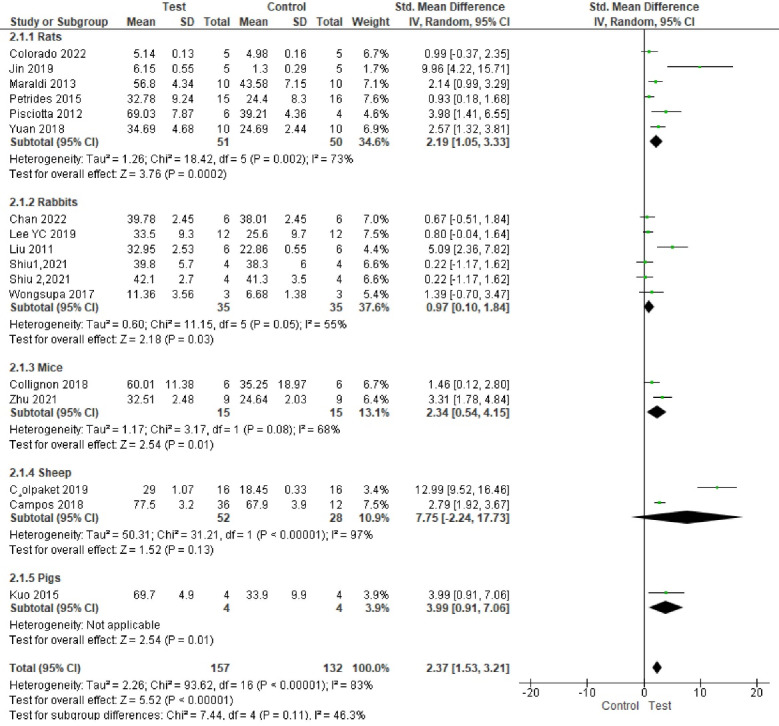
Forest plot for new bone area measures stratified by animal type

**Figure 9:**
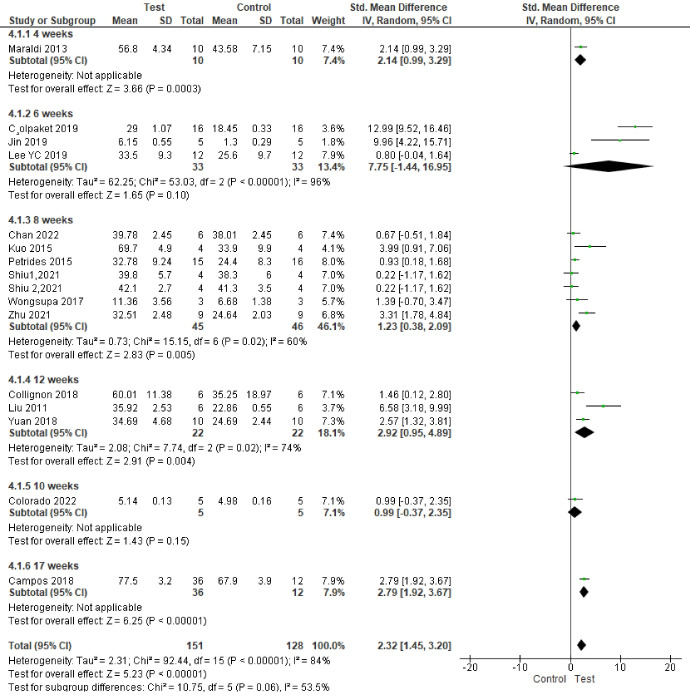
Forest plot for new bone area measures stratified by final follow-up

There were no significant differences in final follow-up and sample types. The most positive impact on bone regeneration occurred in groups where the final follow-up was six weeks, and the sheep were used as samples (Figures 8 and 9). The outcome of sample types subgroup analysis without considering the subgroups including less than two studies was as follows: The biggest SMD occurred in the rat subgroup (SMD = 2.19 [95% CI: 1.05‒3.33], studies = 6), and the smallest SMD occurred in the rabbit subgroup (SMD = 0.97 [95% CI: 0.10‒1.84], studies = 5) (Figure 6).

###  Analysis based on new bone volume percentage

**Figure 10:**
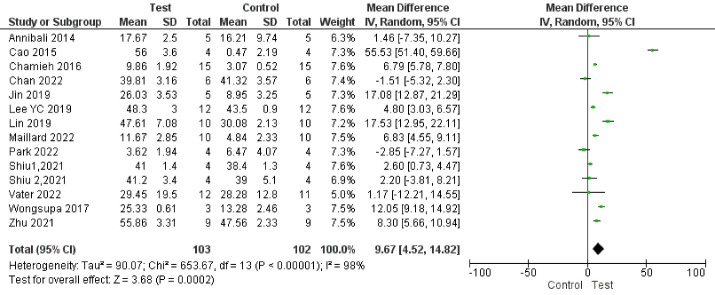
Forest plot for new bone volume measures

**Figure 11:**
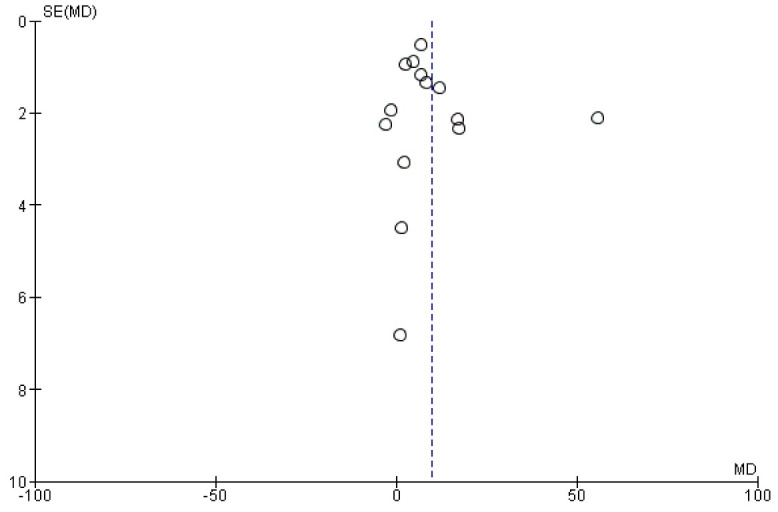
Funnel plot for new bone volume measures

Fourteen studies reported results based on new bone volume percentage.^66,67,68-70,72-75,77,80,83,87^ Study results were highly heterogeneous. Bone formation was significantly enhanced in the test groups compared to the control groups (*P* < 00001, SMD = 1.85, 95% CI: 0.85‒2.85; participants = 205; studies = 14; I^2^ = 84%) (Figures 10 and 11). All the studies showed a net positive effect of DPSCs therapy on bone treatment outcomes. However, two study subgroups^77,83^ reported a negative effect compared to the control groups.

**Figure 12:**
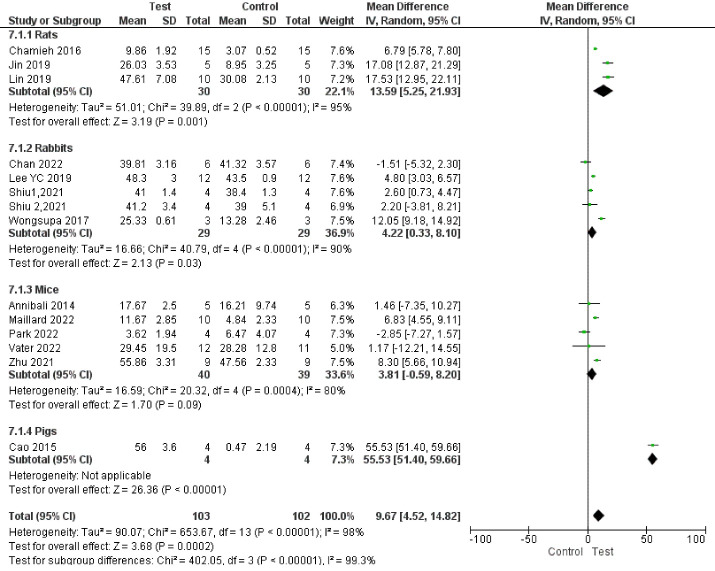
Forest plot for new bone volume measures stratified by animal type

**Figure 13:**
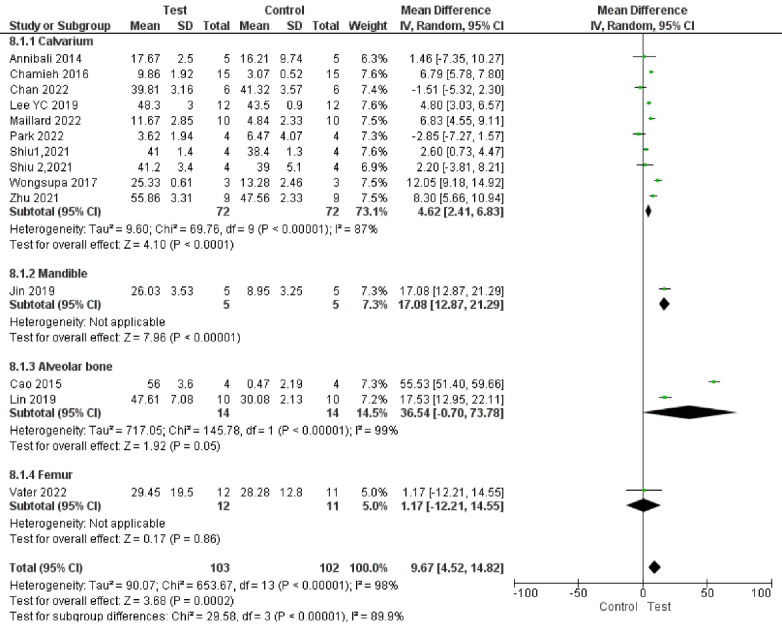
Forest plot for new bone volume measures stratified by defect type

**Figure 14:**
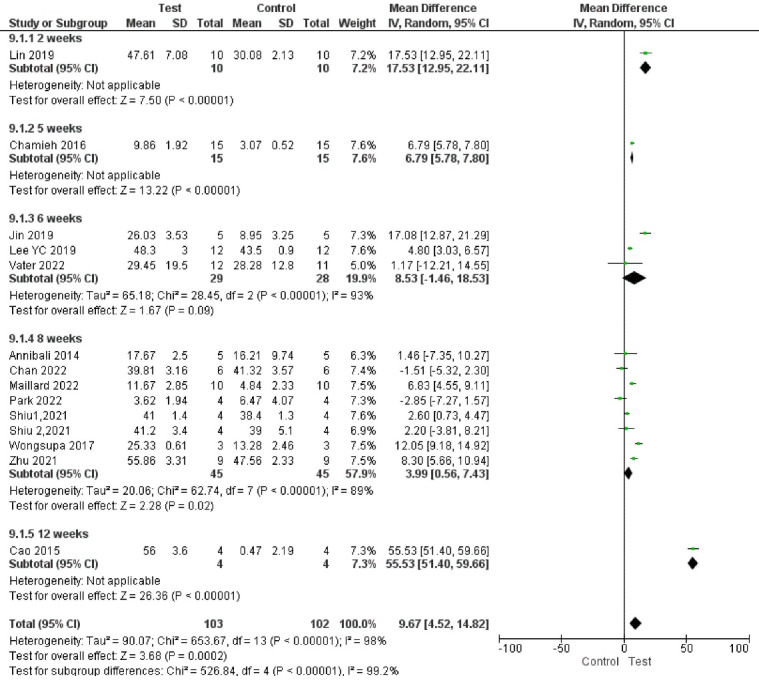
Forest plot for new bone volume measures stratified by the final follow-up

**Figure 15:**
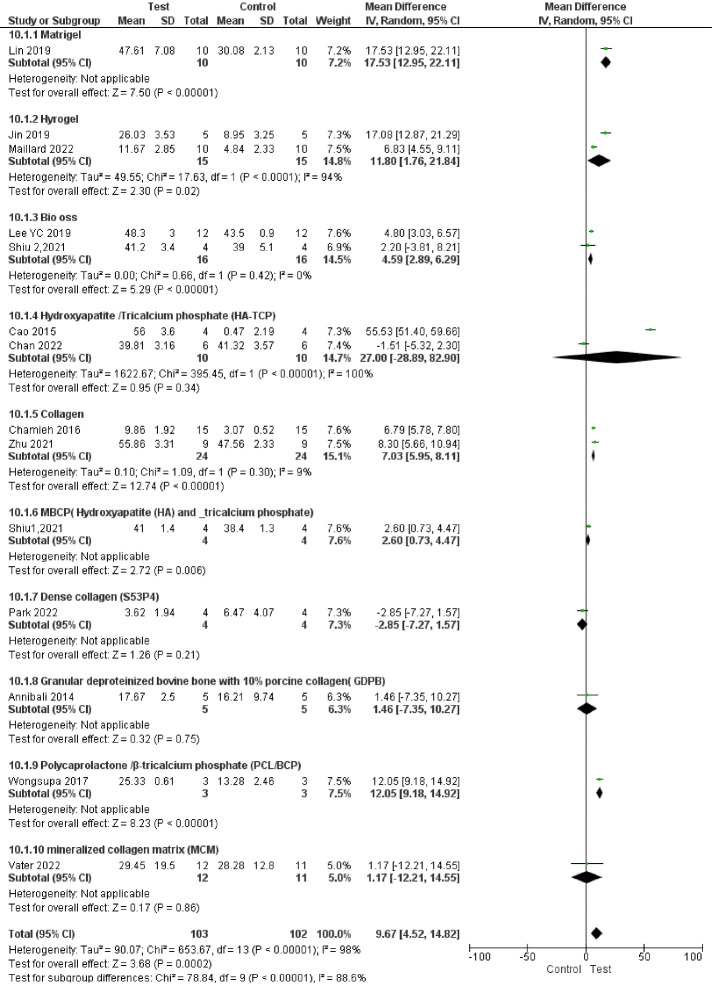
Forest plot for new bone volume measures stratified by scaffold type

Subgroup analyses showed a significant difference in bone regeneration with all the subgroups. However, all the subgroups had only one to two studies, indicating low statistical power. In addition, subgroup analyses showed high heterogeneity (Figures 12, 13, 14, and 15).

Subgroup analyses showed that the most positive impact on bone regeneration occurred in groups where bone defects were created in the alveolar bone, and the pigs were used as samples (Figures 12 and 13). More precisely, the outcomes of subgroup analyses without considering subgroups including less than two studies were as follows: The SMD in the alveolar bone defect subgroup (SMD = 8.48 [95% CI: -4.03‒20.96], studies = 2) was higher than that in the calvarium bone defect subgroup (SMD = 1.58 [95% CI: 0.47‒2.69], studies = 10) (Figure 13). In the sample type subgroups, the SMDs in the rat, rabbit, and mice subgroups were 4.01 ([95% CI: 2.99‒5.03], studies = 3), 1.15 ([95% CI: -0.16‒2.45], studies = 5), and 0.94 ([95% CI: -0.36‒2.24], studies = 5), respectively (Figure 12).

The most significant positive impact on bone regeneration occurred in groups where the final follow-up was 12 weeks, and hydroxyapatite/tricalcium phosphate (HA-TCP) was used as a scaffold (Figures 14 and 15).

###  Reporting biases

There was a possibility of bias due to the small number of studies. Also, the funnel plots of the new bone area and new bone volume indicated an asymmetrical shape. The asymmetrical shape might have been caused by publication bias, study heterogeneity, and methodological anomaly (Figures 5 and 11).

## Discussion

 Bone tissue engineering by DPSCs has been the subject of many studies as a method that could have a promising future in alveolar ridge reconstruction. However, despite many advances in this field, the high heterogeneity of studies and the few studies with complete statistical data make high-power statistical analysis impossible and the clinical application and effectiveness of stem cell utilization unclear.

This meta-analysis evaluated the impact of tissue engineering by DPSCs on bone regeneration based on the new bone volume and new bone area formation. Two previous systematic reviews^6,88^ mentioned a positive impact of tissue engineering by DPSCs on bone regeneration based on qualitative data. The present study is the first to evaluate the effect of tissue engineering by DPSCs on bone regeneration based on quantitative data.

 According to this review, DPSCs and scaffold complexes significantly increase bone regeneration. Clinical diversity and high methodological heterogeneity should be considered in the interpretation of the meta-analysis. Analyses were performed in the subgroups of sample type, scaffold type, final follow-up and defect types. Although heterogeneity decreased in the majority of subgroup analyses, a few studies in subgroups caused the low statistical power of meta-analysis. Nonetheless, the use of DPSCs and scaffold caused a significant increase in the reconstruction of bone defects.

In addition, it is necessary to pay attention to this point that this meta-analysis has not reviewed the impact of other factors, such as growth factors, on bone regeneration. Bone-related factors, gene transfection, and gene overexpression enhance the bone regeneration potential of DPSCs.^5,8^ However, meta-analysis was impossible due to the high heterogeneity of methodology in the studies examining these factors’ impact on bone tissue engineering. Therefore, we can expect a more significant amount of bone regeneration by DPSCs with the application of growth factors, gene transfection, and gene overexpression.

Using growth factors such as tetrahydroxystilbene glucoside,^69^ rhBMP-2,^78^ and osteogenic culture medium^48^ increased bone regeneration. In addition, overexpression of SIRT1, Runx2, EphrinB2, and DPSCs derived from PN3 Wnt1-CRE-Rosa^Tomato^ mouse molar in separate studies^51-53^ showed a significant increase in bone regeneration.

Compared to other stem cells, studies comparing the ability of DPSCs to bone marrow MSCs did not show a significant difference in bone regeneration.^47,50,66,89^ However, evaluation of the osteogenic potential of adipose tissue-derived stem cells^67^ and amniotic fluid stem cells^76^ showed a significant increase in bone regeneration compared to DPSCs.

Overall, using DPSCs with appropriate scaffold, growth factor, and gene therapy will result in the maximum bone regeneration percentage. Finally, as mentioned in previous systematic reviews, bone tissue engineering can be expected to result in a favorable clinical outcome.^6,88^

Few clinical studies examined bone reconstruction in Mansfield.^38-40,43,90,91^ Most of these studies have reported new bone regeneration based on probing depth and clinical attachment loss. Future clinical trials should also evaluate the extent of bone regeneration in other ways, such as micro-computed tomography.

 Future research should concentrate on humans or samples closer to humans, such as dogs and sheep, than on mice and rats. The results should be in the form of statistical data such as bone volume, trabecular number, bone mineral density, and mineral content.

 The new bone formation could include maxillary or mandibular bone defects rather than cranium or subcutaneous ones. Future research should compare the effect of different growth factors, scaffolds, and gene overexpression on bone regeneration.

## Conclusion

 Bone tissue engineering by DPSC is one of the promising ways for bone regeneration in the future. This study was designed in response to the question of whether the current clinical studies quantitatively indicate the ability of DPSC to regenerate bone properly. In this review article, the meta-analysis conducted on the results of the studies showed a significant increase in the amount of bone regenerated by DPSCs. It also showed a ‘large’ effect size by DPSC on bone regeneration. However, more studies in the future will provide the possibility of meta-analysis with more power. Furthermore, to achieve the best method of transplanting DPSCs in bone tissue engineering, future studies should compare the effects of growth factors, types of biological scaffolds, and other factors affecting bone regeneration by DPSCs. Therefore, more preclinical and clinical studies should be conducted in this field to overcome the clinical challenges of tissue engineering by DPSCs.

## Funding

 This study was supported by a grant [No: 456197] from Birjand University of Medical Sciences (BUMS), Birjand, Iran.

## Ethics Approval

 Not applicable.

## Competing Interests

 There are no conflicts of interest.
